# Delayed-onset radiculopathy caused by a retropulsed bone fragment after percutaneous kyphoplasty: report of four cases and literature review

**DOI:** 10.1186/s12891-022-05472-w

**Published:** 2022-06-02

**Authors:** Yi-Hsuan Lee, Po-Quang Chen, Chung-Ting Wu

**Affiliations:** 1 Department of Orthopaedics, Taipei Tzu Chi Hospital, Buddhist Tzu Chi Medical Foundation, No. 289, Jianguo Rd., Xindian Dist., (R.O.C.), Taipei City, 231 Taiwan; 2 Department of Orthopaedic Surgery, National Taiwan University Hospital, Chung Shan S. Rd., Zhongzheng Dist., (R.O.C.), Taipei City, 100225 Taiwan; 3 Kang-Ning General Hospital, No. 26, Ln. 420, Sec. 5, Cheng Gong Rd., Neihu Dist., (R.O.C.), 114 Taipei, Taiwan

**Keywords:** Osteoporosis, Kyphoplasty, Retropulsed bone fragment, Radiculopathy, Complication

## Abstract

**Background:**

Vertebral compression fractures caused by osteoporosis are common in elderly patients and are often encountered by clinical physicians. Percutaneous balloon kyphoplasty (PKP) is widely accepted as a minimally invasive procedure for effectively relieving pain and correcting deformities, but complications may occur. Radiculopathy with a delayed onset caused by a retropulsed bone fragment has not been adequately described in the literature. Thus, this article presents a case report of four cases of retropulsed bone fragment-related radiculopathy after PKP.

**Case presentation:**

In this article, we reported that four out of 251 patients developed radiculopathy after PKP between January 2012 and January 2019 despite experiencing substantial improvements in back pain. All patients with radiculopathy were female and diagnosed with osteoporosis, and their ages ranged from 68 to 89 years. Radiculopathy occurred from 2 to 16 weeks after PKP. All four patients underwent another operation (posterior decompression and instrumentation). Three patients recovered completely, and one died of postoperative intracranial haemorrhage. A detailed imaging study with pre- and postoperative magnetic resonance imaging (MRI) revealed that retropulsed bone fragments that impinged on the corresponding root after PKP were responsible for this complication, and all four patients developed a disrupted posterior vertebral rim preoperatively. No leakage of cement or pedicle track violations were observed.

**Conclusion:**

Although PKP is a safe and effective treatment for painful osteoporotic vertebral compression fractures, a risk of catastrophic neurological injury remains. Radiculopathy with delayed onset caused by a retropulsed bone fragment after kyphoplasty is rare and challenging to treat, and the integrity of the posterior vertebral cortex should be carefully evaluated preoperatively to prevent this complication.

## Background

Osteoporotic vertebral compression fractures (VCFs) constitute a global burden that temporarily or permanently affects millions of elderly people worldwide. Percutaneous balloon kyphoplasty (PKP) is a surgical technique for the treatment of osteoporotic VCFs and is widely accepted as an effective minimally invasive procedure [1, 2]. Despite encouraging clinical outcomes, some related complications, such as cement extravasation, emboli, and adjacent level fractures, have been reported following the procedure [1]. However, radiculopathy caused by a retropulsed bone fragment has never been reported. Herein, we present a series of four cases of retropulsed bone fragment-related radiculopathy after PKP, and the mechanism resulting in its occurrence is discussed.

## Case presentation

From January 2012 to January 2019, we subjected 251 patients to PKP. In this case report, we identified four patients who, despite experiencing substantial improvements in back pain, developed radiculopathy after PKP. Delayed-onset radiculopathy was noted from 2 to 16 weeks after PKP. The baseline characteristics of the patients, their clinical manifestations, and their courses of treatment are presented below (Table 1).

**Table 1:** Patient characteristics

	Patient 1	Patient 2	Patient 3	Patient 4
Age	77	78	68	89
Sex	female	female	female	female
Level	T12	L2	T12	L2
Etiology	osteoporosis	osteoporosis	osteoporosis	osteoporosis
Procedure	kyphoplasty	kyphoplasty	kyphoplasty	kyphoplasty
Cement volume	8.0 cc	4.0 cc	4.5 cc	5.0 cc
Duration until radiculopathy	8 weeks	8 weeks	16 weeks	2 weeks
Symptoms	Groin pain	Anterior thigh pain	Groin pain	Anterior thigh pain
Treatment	PD + PI	1^st^OP: PD 2^nd^OP: PD + PI	PD + PI	PD + PI
Outcomes	Full relief	Mortality (ICH)	Full relief	Full relief

*PD* posterior decompression

*PI* posterior instrumentation

*ICH* intracerebral hemorrhage

This study was approved by the institutional review board of our medical centre. Additionally, all the patients consented to publication of their clinical data.

### Case 1

**Fig. 1:**
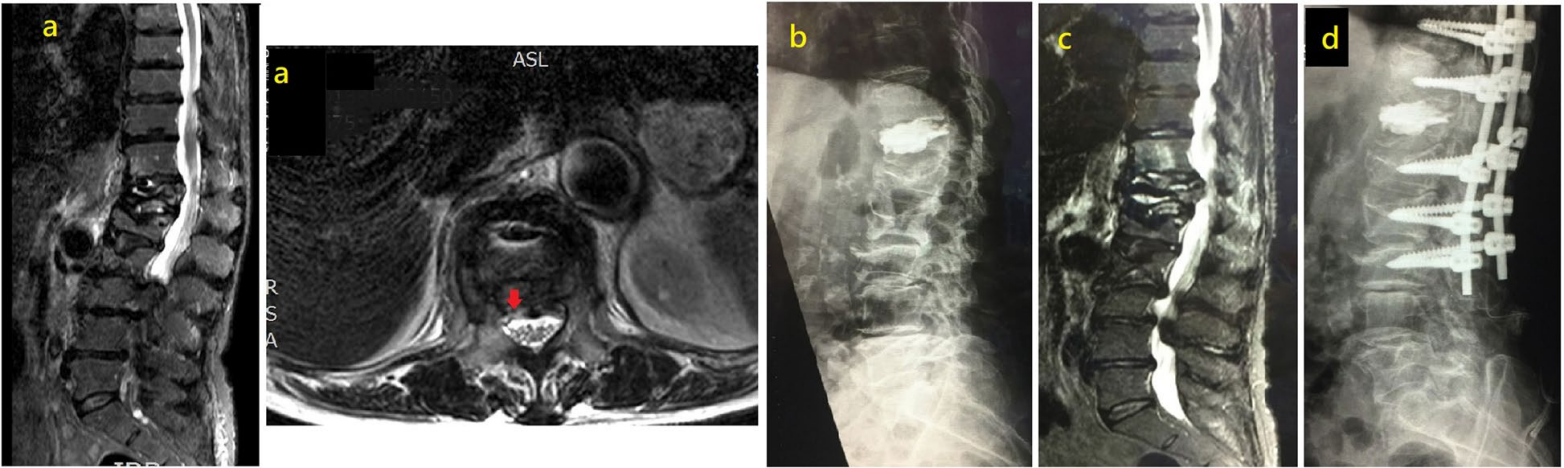
MRI showing a 77-year-old female with T12 compression fracture, disrupted posterior vertebral rim was identified (red arrow) (**a**). Lateral view of lumbar spine X-ray of a T12 VCF underwent PKP with 8.0 c.c. bone cement (**b**). Patient developed right T12 radiculopathy 2 months after PKP, MRI showing spinal canal compromised by a retropulsed bone fragment (**c**). We performed posterior decompression (PD) of T12 with instrumentation from T10-L3 (**d**)

A 77-year-old female with a T12 VCF underwent PKP with 8.0 cc of bone cement. Her back pain improved substantially after PKP; however, 2 months after PKP, she developed right T12 radiculopathy. Magnetic resonance imaging (MRI) demonstrated spinal canal compromise caused by a retropulsed bone fragment. We performed posterior decompression (PD) at the T12 level with instrumentation from T10 to L3, and she experienced relief of all of her symptoms after this procedure (Fig. 1).

### Case 2

**Fig. 2:**
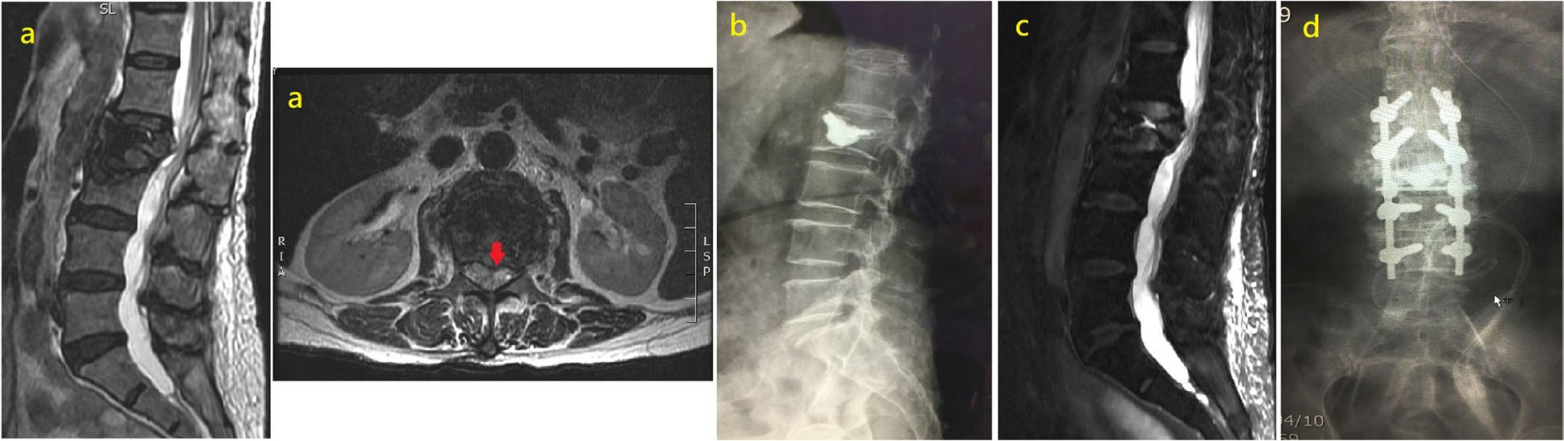
MRI of L2 compression fracture with posterior vertebral cortex disruption before PKP (red arrow) (**a**). We performed L2 PKP with 4.0 c.c. bone cement (**b**). MRI showing spinal canal compromised by a retropulsed bone fragment after balloon kyphoplasty (**c**). L2 level posterior decompression (PD) and T12-L4 instrumentation (PI) with pedicle screws (**d**)

A 78-year-old female with an L2 VCF underwent PKP with 4.0 cc of bone cement. She complained of left anterior thigh pain 2 months after surgery despite symptomatic relief of her back pain, and MRI showed spinal canal compression at the L2 level. Posterior decompression was first performed, and partial symptom relief was achieved; however, her symptoms recurred, and MRI showed further vertebral body collapse and spinal canal restenosis 4 months after decompression. We performed L2-level posterior decompression (PD) and T12-L4 instrumentation (PI) with pedicle screws. Unfortunately, the patient died due to immediate postoperative intracranial haemorrhage 7 days after surgery (Fig. 2).

### Case 3

**Fig. 3:**
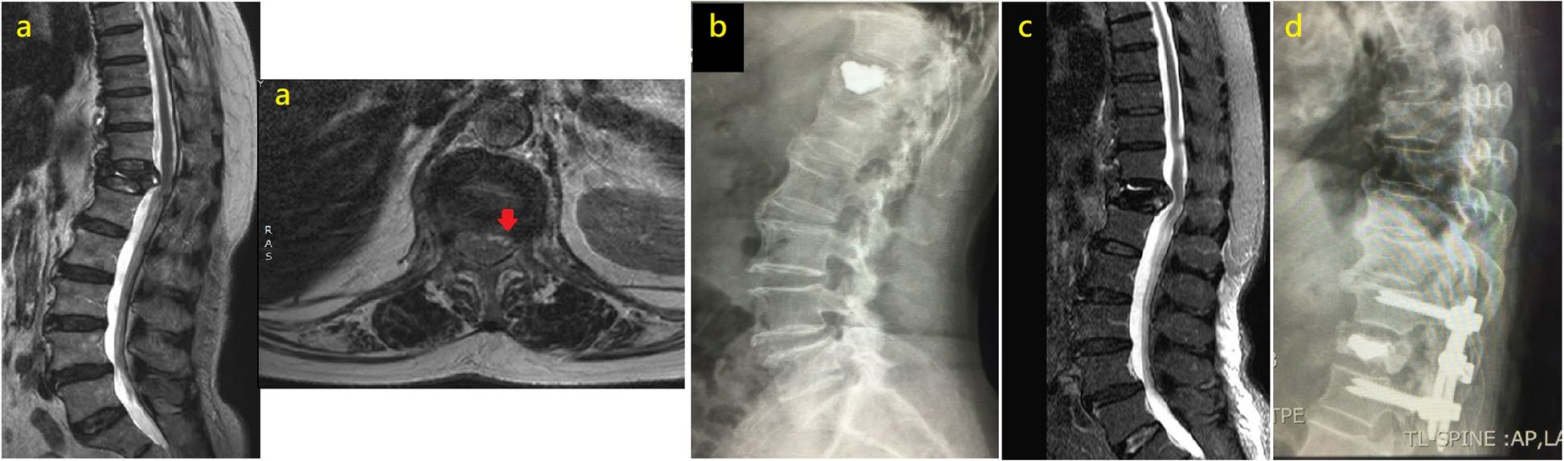
MRI showing T12 compression fracture with posterior vertebral rim disruption (red arrow) (**a**). T12 PKP was performed (**b**). Patient complained of right groin pain (L1 dermatome) 4 months after PKP, MRI proving spinal canal compression at T12 level (**c**). We performed T11-L1 level posterior decompression and instrumentation (**d**)

A 68-year-old female with a T12 VCF underwent PKP and complained of right groin pain 4 months after surgery. MRI showed spinal canal compression at the T12 level. We performed decompression with T11-L1 PI, and her symptoms and signs resolved soon afterwards (Fig. 3).

### Case 4

**Fig. 4:**
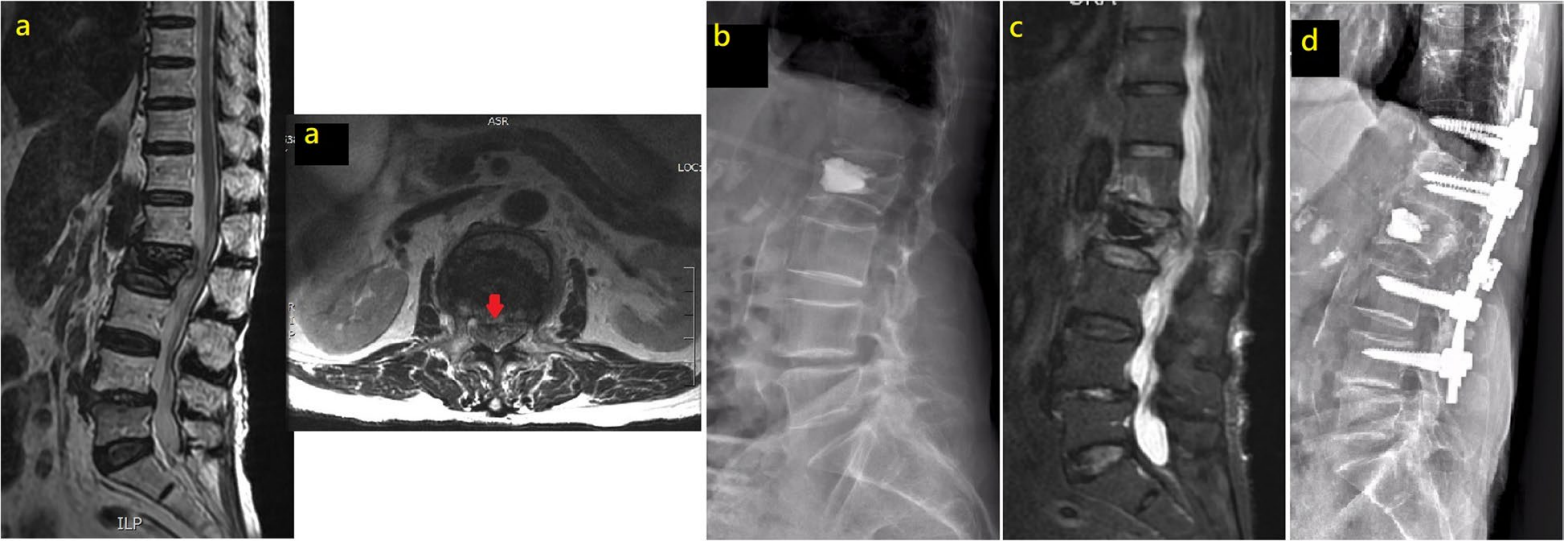
MRI of L2 compression fracture with posterior vertebral rim disruption (red arrow) (**a**). L2 PKP was performed, left L2 radiculopathy developed 2 weeks after surgery (**b**). MRI showing further protrusion of the posterior vertebral fragment posteriorly (**c**). Surgical decompression and T12-L4 instrumentation were performed (**d**)

An 89-year-old female with an L2 VCF underwent PKP and developed left L2 radiculopathy 2 weeks after surgery. MRI revealed further protrusion of the posterior vertebral fragment posteriorly. Surgical decompression and T12-L4 PI were performed, and full symptom relief was observed after the procedure (Fig. 4).

Herein, we report four cases of radiculopathy with a delayed onset after PKP, despite substantial improvements regarding back pain. A detailed imaging study with postoperative MRI revealed further collapse of the posterior vertebral body and retropulsed bone fragments invading the root and spinal canal, particularly in patients with a disrupted posterior vertebral cortex preoperatively. Four patients with radiculopathy underwent PD and PI, and full symptom relief was achieved in three patients. One patient died due to postoperative ICH.

## Discussion and conclusions

Compression fractures caused by osteoporosis are common in elderly patients and are often encountered by clinical physicians. In outpatient or emergency departments, these patients are first subjected to conservative management with bed rest, oral analgesics (opioid analgesics, nonsteroid anti-inflammatory drugs and muscle relaxants) and immobilization with thoracolumbosacral orthosis (TLSO). Further osteoporosis assessment (bone mineral density (BMD) calcium and vitamin D level) and anti-osteoporotic medications are administrated at the following outpatient department visit. If patients continue to experience unbearable pain, the patients are subjected to inpatient care consisting of bed rest, IV analgesics (morphine, parecoxib or propacetamol) and other symptomatic treatment. Patients with neurological deficits or who remain incapacitated or bedridden due to severe back pain are subjected to emergent CT or MRI scans; if an imaging study shows spinal cord/root compression, emergent neural decompression and posterior instrumentation is performed, and kyphoplasty is not considered. For patients with neither neurological deficits nor spinal cord/root compression on MRI images, PKP has been recognized as a surgical procedure for back pain relief because it can augment the collapsed vertebral body and correct deformities of sagittal alignment [2]. Although kyphoplasty is a minimally invasive, percutaneous technique, cement augmentation of vertebral fractures is associated with intra- and postoperative complications [1]. Taylor et al. [3] conducted a meta-analysis and reported the complications of kyphoplasty, including extravertebral cement leakage (8.1%), secondary adjacent vertebral compression (9.4%), pulmonary emboli (0.17%), and perioperative mortality (0.13%).

Among all the complications caused by kyphoplasty, radiculopathy has rarely been reported. Patel et al. [4] reported 14 patients who exhibited neurological deficits after a vertebral cement augmentation procedure (4 underwent vertebroplasty, and 10 underwent kyphoplasty). The observed deficits in six patients were caused by cement extravasation (by vertebroplasty in two cases and by kyphoplasty in four cases), and those in eight patients were due to a retropulsed bone fragment at the treated level or adjacent fracture.

In this study, we found that four out of 251 patients developed radiculopathy after kyphoplasty despite achieving substantial relief of their back pain. The symptoms developed two weeks to four months after surgery. After conducting detailed preoperative MRI analyses and reviewing the previously published literature [1, 4–11] and our experiences, we proposed the following four possible causes of radiculopathy after kyphoplasty:

1. **Retropulsed bone fracture**
  Preoperative posterior vertebral cortex integrity is the keystone of complications such as radiculopathy. Preoperative MRI of the four patients who developed radiculopathy after kyphoplasty revealed a disruption of the posterior vertebral rim, whereas the other 247 patients presented with an intact posterior vertebral rim on MRI. It can be hypothesized that during the surgical procedure, the inflated balloon pushes the posterior vertebral bone fragment backwards, which subsequently compresses the spinal canal or neural foramen. The symptoms exhibited by these patients vary depending on which vertebral level is compressed. Instead of classic sciatic pain, lower abdominal and inguinal pain are the most common symptoms; therefore, delayed diagnosis is highly likely, and patients may initially seek aid from urologists or gastroenterologists.
2. **Refracture**
  Kim and Lavelle both reported that refracture of previously treated vertebrae could cause compression of the nerve root [5, 6]. The incidence of refracture is approximately 10–12%, and refracture develops at an average of 3 months after surgery. The predisposing factors of refracture include intravertebral cleft (osteonecrosis) and non-polymethylmethacrylate (PMMA)-endplate contact. Li et al. [7] reported three cases of refracture, and one patient experienced recurrent back pain and weakness in both legs. MRI revealed refracture, cement fragmentation, and neural canal encroachment.
3. **Cement leakage**
  The risk of neurological compression due to foraminal or intracanal leakage of cement is low but has been reported. Hsieh et al. [8] reviewed 3175 patients who underwent vertebroplasty and found that only four patients exhibited neurological deficits (0.26%) caused by cement leakage. Majd [9] reported a cement leakage incidence of 10.6%, and one of 222 patients (0.45%) experienced L1 radiculopathy after kyphoplasty due to cement extravasation and recovered after selective nerve block and rehabilitation.
4. **Inferior endplate fracture**
  Different VCF patterns may have a strong influence on newly developed radiculopathy and back pain. Kim et al. [10] analysed 59 patients with vertebral fractures by MRI based on the classification proposed by Kanchiku [12], which categorizes fractures into three types: superior, middle, and inferior fractures. The authors concluded that inferior-type fractures are more related to the development of radiculopathy, which can be treated by bone cement augmentation due to fracture reduction and stabilization. However, in our opinion, preoperative radiculopathy is a relative contraindication for kyphoplasty, and the optimal treatment remains under debate.

**Fig. 5:**
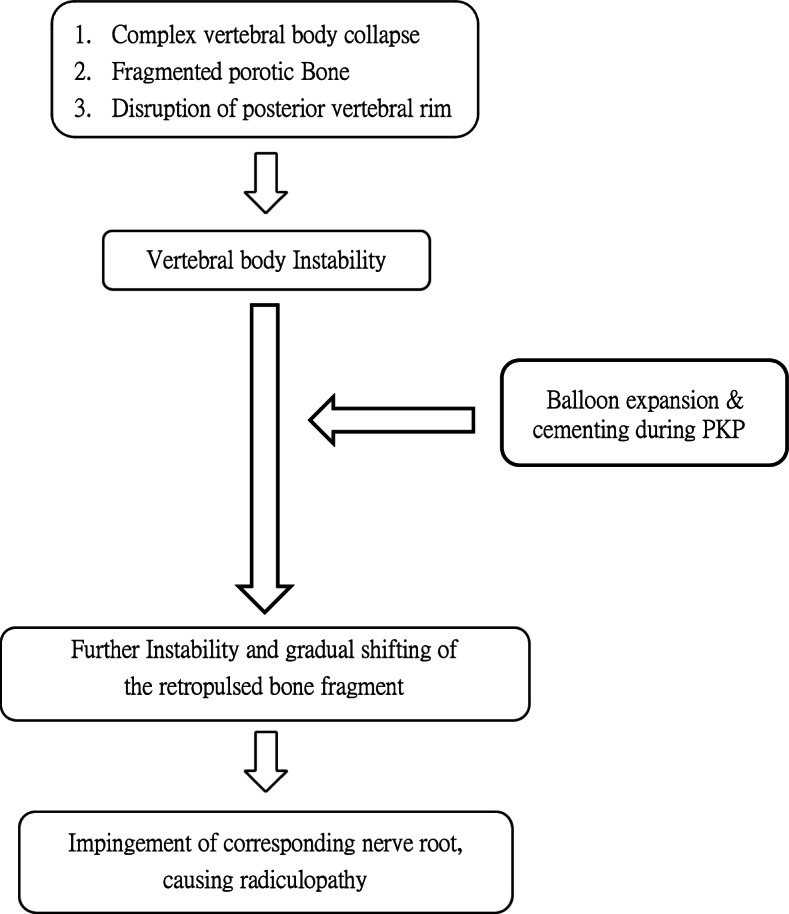
Mechanism of radiculopathy caused by a retropulsed bone fragment after percutaneous kyphoplasty

In this article, we report four cases of radiculopathy after kyphoplasty. To the best of our knowledge, this study constitutes the first investigation of the aetiology of radiculopathy after PKP. We retrospectively reviewed the preoperative MRI scans of all the patients and found that all patients exhibited a disrupted posterior vertebral rim. Due to a lack of intact posterior confinement, the bone fragment might have been pushed backwards by balloon inflation during PKP and might have caused nerve root or spinal canal compression (Fig. 5). This serious complication caused us to reflect on the decision-making process during the treatment of these patients. In our practice, we only perform kyphoplasty instead of vertebroplasty, because of the resulting better kyphotic angle correction and lower rate of cement extravasation. However, because a balloon tamp is not used during vertebroplasty, it may be associated with a low risk of fragment retropulsion, which may be an appropriate option in these patients. Hiwatashi [11] reported 21 patients with compression fractures and retropulsed fragments who were treated with vertebroplasty; however, the mean size of the retropulsed fragments increased from 4.2 mm to 4.4 mm, and no patients developed new neurological symptoms.

We hypothesize that when treating VCFs, conservative treatment, which includes TLSO, bed rest, and oral analgesic agents, should first be attempted, and even teriparatide has been suggested to be effective [13, 14]. In patients who are refractory to conservative treatment, if VCFs do not invade the posterior rim and no spinal canal stenosis is observed, kyphoplasty may be an alternative option for achieving stabilization and pain relief. If posterior rim breach or spinal canal compromise is observed, kyphoplasty in combination with posterior decompression and instrumentation might be an effective and safe option [15–17] and is also the current standard procedure in our practice. The results of this procedure are satisfactory. However, once the spinal canal or neural foramen is compromised by a retropulsed bone fragment after PKP, the outcomes of conservative treatment for this complication are usually unsatisfactory. In our experience, the symptoms can be successfully relieved only by surgical decompression, neurolysis, and posterolateral fusion with the pedicle screw system. Despite our favourable results, this study has some limitations. The proposed mechanism of radiculopathy caused by retropulsed fragments is presumptive and based on MRI scans after PKP. Experimental data, including the accurate size of retropulsed fragments and the correlation between neurological signs and the percentage of compromised spinal canals, are lacking. Another limitation of our study is the relatively small case number and short follow-up period. In the future, clinical trials for comparative studies with larger case numbers and longer periods are needed.

In conclusion, PKP is mostly a safe and effective treatment for painful osteoporotic vertebral compression fractures. Although serious complications are uncommon, a catastrophic neurological injury, such as the condition reported in this article, remains possible. Delayed-onset radiculopathy after PKP is a rare but challenging condition. Preoperative posterior vertebral cortex integrity should be carefully evaluated to determine the optimal surgical procedure. Once this complication is encountered, an MRI scan needs to be performed to determine the causes and management methods. However, if cement causes artefacts on MRI images, CT scans will be an alternative option. In our experience, prompt surgical decompression and posterior instrumentation provide an option for symptom relief and prevention of further vertebral collapse. However, the patient’s general condition and comorbidities should also be considered.

## Data Availability

The data that support the findings of this study are available from the *Cathay General Hospital Ethical Committee*, but restrictions apply to the availability of these data, which were used under licence for the current study and are thus not publicly available. However, the data are available from the authors upon reasonable request and with permission from the *Cathay General Hospital Ethics Committee*.

## Abbreviations

VCF: Vertebral Compression Fracture
PKP: Percutaneous Balloon Kyphoplasty
MRI: Magnetic Resonance Imaging
CT: Computed Tomography
PD: Posterior Decompression
PI: Posterior Instrumentation
DIC: Disseminated Intravascular Coagulation
TLSO: Thoracolumbosacral Orthosis
PMMA: Polymethylmethacrylate
